# Immunoglobulin M Nephropathy in a Patient with Wilson’s Disease

**DOI:** 10.7759/cureus.929

**Published:** 2016-12-13

**Authors:** Zain Ul Abideen, Zoya Sajjad, Asna Haroon Khan, Nadira Mamoon, Muhammad Bilal, Khaja Hameeduddin Mujtaba Quadri

**Affiliations:** 1 Nephrology and Renal Transplant, Shifa International Hospital, Islamabad, Pakistan; 2 Internal Medicine, Shifa International Hospital, Islamabad, Pakistan; 3 Pathology, Shifa International Hospital, Islamabad, Pakistan

**Keywords:** immunoglobulin m nephropathy, nephrotic syndrome, wilson's disease

## Abstract

Immunoglobulin M nephropathy (IgMN) is characterized by the deposition of immunoglobulin M in a dominant distribution in the renal glomeruli. Primary immunoglobulin M nephropathy is diagnosed after consistent light microscopy (LM), immunofluorescence (IF), electron microscopy (EM) results, and exclusion of known systemic disorders causing immunoglobulin M deposition in the glomeruli. The secondary disease has been reported with a few conditions though it has never been reported with any primary disease of the liver. We report the case of an adolescent male patient who presented with nausea, vomiting, diarrhea, and worsening anasarca. He was found to have nephrotic-range proteinuria that did not respond to conventional corticosteroid treatment. He was subjected to a renal biopsy which revealed a diagnosis of immunoglobulin M nephropathy. His liver function tests were deranged and an ultrasound scan of the abdomen revealed a coarse irregular liver. Workup revealed elevated urine copper excretion and a low ceruloplasmin level. He was diagnosed as a case of Wilson’s disease and started on penicillamine and pyridoxine. He was also started on intravenous cyclophosphamide for the corticosteroid-resistant nephrotic syndrome to which he responded remarkably well. His edema settled, proteinuria resolved, and liver functions normalized. Currently, he is in remission and enjoying good health. To the best of our knowledge, we report the first known association between IgM nephropathy and Wilson’s disease. It is presently not clear if causation can necessarily be established. This may be the result of defective IgM clearance by the liver or an altered metabolism of the antibody or immune complexes, as with hepatic-associated immunoglobulin M (IgM) nephropathy. Further studies are needed to elucidate the exact mechanism of this disease.

## Introduction

Immunoglobulin M nephropathy (IgMN) is a glomerulopathy characterized by mesangial hypercellularity on light microscopy (LM) and immunoglobulin M (IgM) deposition on immunofluorescence (IF) and electron microscopy (EM) [1]. It was first described in the 1970s and since then has undergone considerable controversy as to whether it is a separate entity [2]. Patients have a variety of presentations, most commonly, the idiopathic nephrotic syndrome [2]. A few conditions are reported with immunoglobulin M (IgM) nephropathy including systemic lupus erythematosus (SLE), rheumatoid arthritis, Alport syndrome, paraproteinemias, and diabetes mellitus [2]. The mechanism of the disease is unknown, though numerous hypotheses have been put forward, which will be discussed later in the article. We present the case of a young adolescent male, who presented to us with worsening generalized body edema and proteinuria. He had a steroid-resistant disease and underwent a renal biopsy which revealed IgM nephropathy. Subsequently, he responded to three doses of cyclophosphamide and is currently in disease remission. During workup, he was also found to have elevated liver enzymes and a coarse liver echotexture. Workup was done by the gastroenterology team which diagnosed him with Wilson’s disease, for which he was started on penicillamine and offered family screening. To the best of our knowledge, IgM nephropathy has never been reported before with Wilson’s disease or a primary disease of the liver. Our case may shed some light on the uncertain mechanism of this glomerulopathy. Informed consent was obtained from the patient for this study.

## Case presentation

A 15-year-old adolescent male from Punjab, Pakistan presented to the gastroenterology clinic with nausea, vomiting, and diarrhea for a week. He vomited two to three times per day, often after a meal. The vomits were not bloody or bilious and contained only food particles. The diarrhea was semi-solid to watery without any mucus or blood. He had these episodes three to four times a day. There was no fever or any abdominal pain. On general physical examination, he had a swollen face, puffy eyes, and peripheral edema. There was pallor. His pulse was 80/minute, blood pressure 120/70 mm hg, respiratory rate 16/minute, he was afebrile and weighed 65 kg. An abdominal examination revealed distention and shifting dullness. There was no visceromegaly. The examinations of the respiratory, cardiovascular, and neurological systems were unremarkable.

His initial laboratory parameters are tabulated in Table 1.

**Table 1:** Laboratory parameters

Investigation	Patient Value	Normal Value
Hemoglobin (g/dL)	14.60 g/dl	13.5 – 17.5 mg/dL
Serum albumin (g/dL)	1.20 g/dL	3.5-4.1 mg/dL
Serum cholesterol (mg/dL)	209mg/dL	Less than 200 mg/dL
Urine protein creatinine ratio on first presentation (mg/mg)	17.10 mg/mg	Less than 0.11 mg/mg for males
Urine protein creatinine ratio after a month of steroid treatment (mg/mg)	8.22 mg/mg	Less than 0.11 mg/mg for males
Urine protein creatinine ratio after treatment with three doses of cyclophosphamide (mg/mg)	0.5 mg/mg	Less than 0.11 mg/mg for males

An ultrasound scan of the abdomen revealed a coarse heterogeneous outline of the liver and moderate ascites. A urine examination revealed three plus proteinuria. He was referred to the nephrology clinic. A urine protein creatinine ratio (PCR) was performed, which estimated about 17 grams of proteinuria per 24 hours. The results are tabulated at various stages of patient treatment. The serum complement levels were within normal range.

Meanwhile, a workup for the chronic liver disease was sent. His markers for viral hepatitis was negative. The workup for autoimmune hepatitis was negative. Anti-nuclear antibody was also negative. The serum ceruloplasmin levels were 9 mg/dL (20-60 mg/dL). The 24-hour urine copper excretion revealed 272 micrograms of copper excreted per day (normally less than 60 micrograms/day). His ophthalmological examination was normal. On the basis of all these parameters, he was diagnosed as a case of Wilson’s disease by the consultant gastroenterologist. There was no history of this disease or related manifestations in the family.

**Figure 1:**
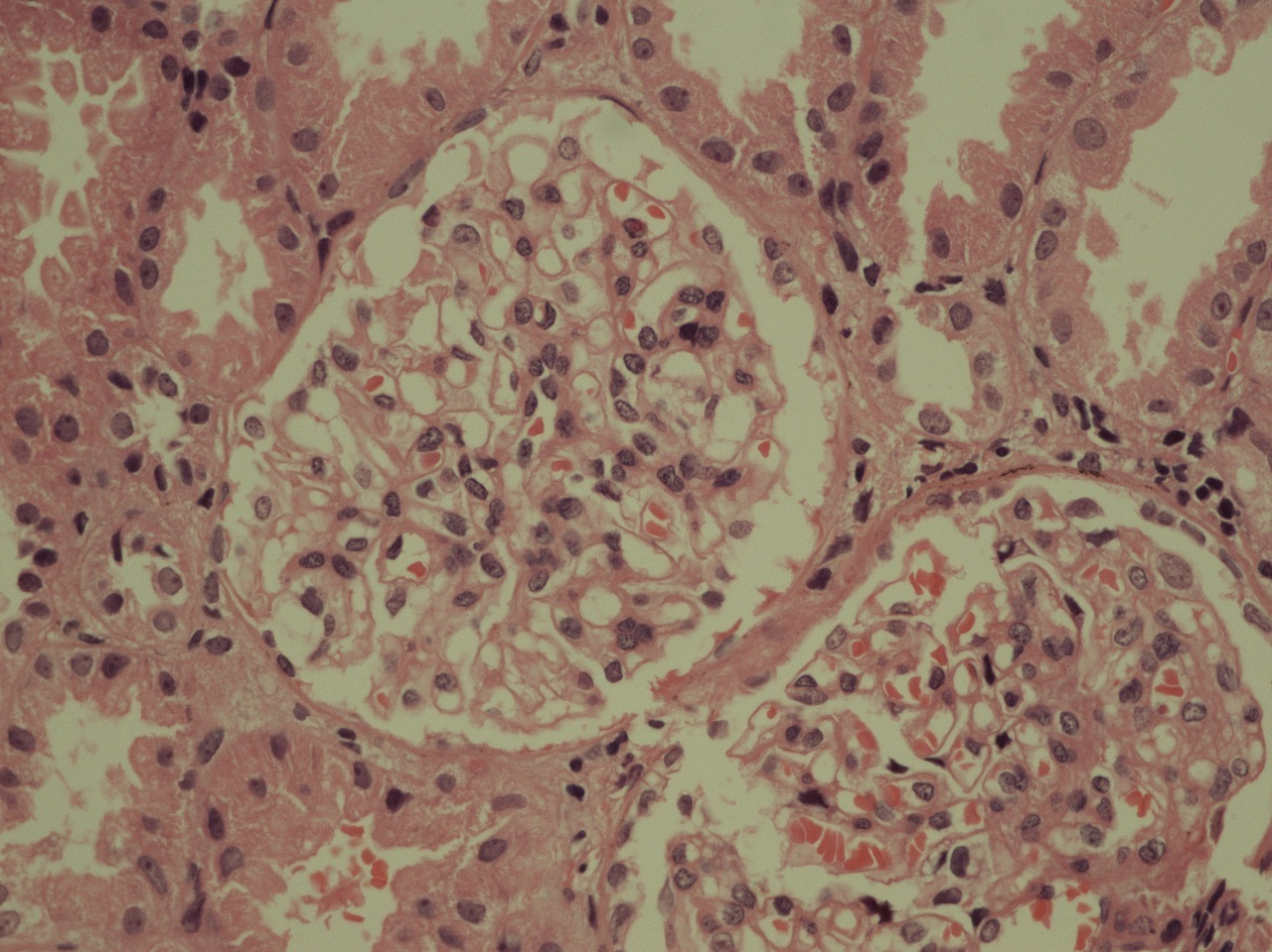
Histopathological image of the renal biopsy as seen under the light microscope Light microscopy revealed focally increased mesangium and cellularity in the glomeruli.

**Figure 2:**
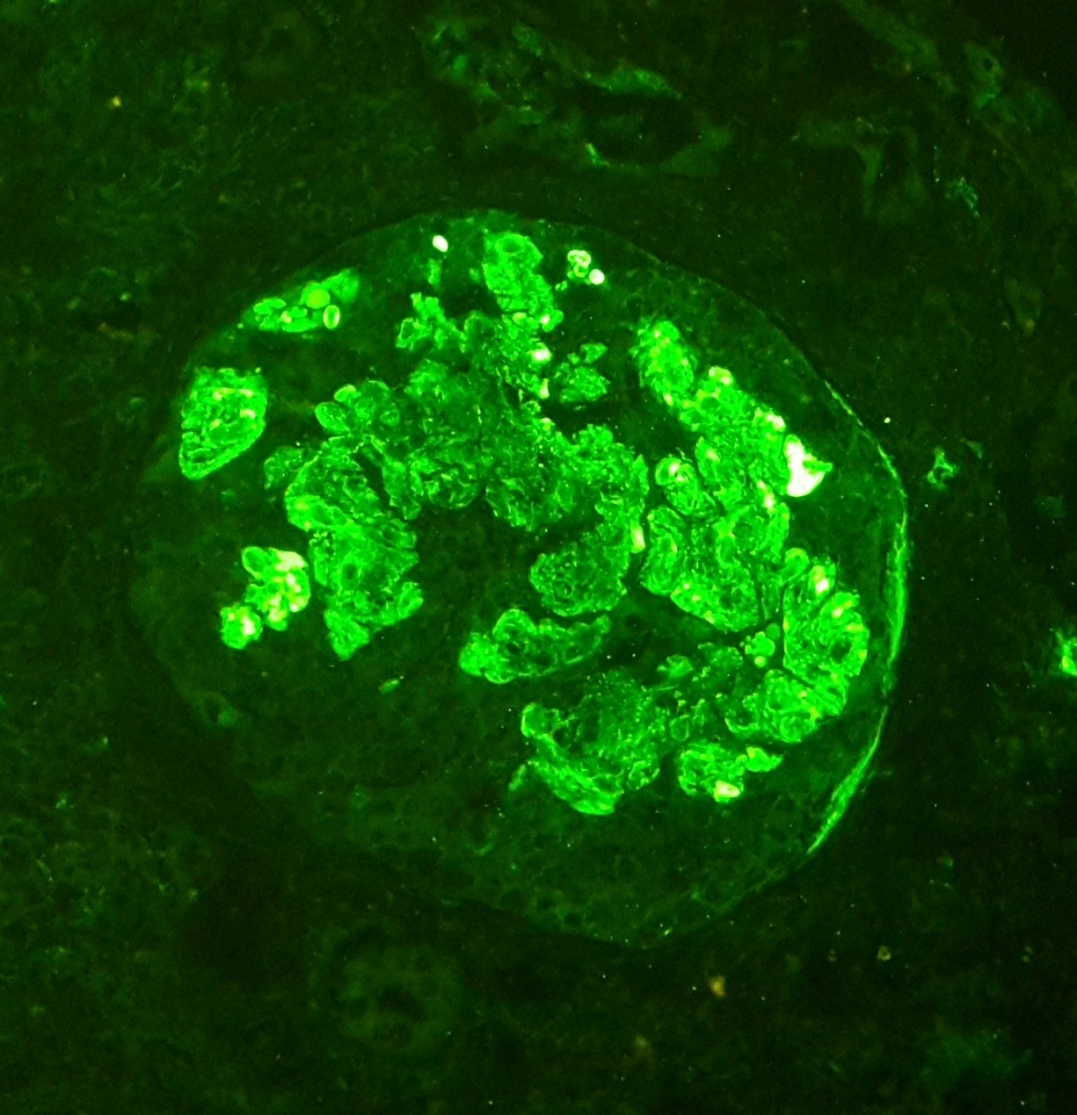
Immunofluorescence image of the renal biopsy specimen Immunofluorescence showed diffuse, 3+ fine granular deposits of IgM in the mesangium.

He was started on tablet penicillamine 250 mg twice daily along with pyridoxine. Meanwhile, he was given a trial of oral prednisolone at 20 mg twice daily for a month and started on spironolactone at 50 mg/day, which was later increased to 100 mg/day. He was initiated on a salt restricted diet and started on atorvastatin 5 mg once daily. On the follow-up visit, he was found to have persistent proteinuria as tabulated. A renal biopsy was performed after informed consent was obtained. Light microscopy (LM) revealed focally increased mesangium and cellularity in the glomeruli (Figure 1). Immunofluorescence (IF) showed 3+ fine granular deposits of IgM in the mesangium (Figure 2).

The patient was then subjected to three doses of intravenous cyclophosphamide separated one month apart. The doses were 300 mg for the first and 400 mg each for the second and third cycle, respectively. Corticosteroids were continued at the same dosage along with atorvastatin at 5 mg daily.

The patient had regular follow-up visits in gastroenterology and nephrology clinics and he showed significant improvement after the third dose of intravenous (IV) cyclophosphamide. The urine PCR declined to 0.5 and the serum albumin rose to 3.8 g/dL. His edema resolved completely and he came off diuretic treatment. A year after these events he developed a relapse of the disease, which was treated successfully with corticosteroids and mycophenolate mofetil. Currently, he is on disease remission with no proteinuria or edema and enjoying good health.

## Discussion

Since its initial description, IgM nephropathy has been described from various parts of the world. Its prevalence ranges between two percent and 18% [2]. A recent study from Europe revealed a frequency of 1.8% [3]. A previous study from Pakistan reported a prevalence of 10.37% from children presenting with idiopathic nephrotic syndrome [4]. There is no consensus on the definition of IgM nephropathy. Most studies, however, have diagnosed this disease in the presence of IgM deposits in the mesangium. The amount of IgM required for diagnosis is again a subject of debate. Some authors have set the threshold to as low as trace positivity, while others have used more stringent criteria with at least a one or two plus positivity. Other immunoglobulins may also be found but their amount should be less than IgM. Complement products may also be found, mostly complement 3 (C3) and C1q [2-3]. LM and EM findings can be variable. In a past study, mesangial matrix expansion was the most common LM finding [1]. Changes consistent with focal segmental glomerulosclerosis may be also be found, while in some cases, glomerular changes may be subtle [2]. Tubulointerstitial and vascular changes may also be found [2]. Our patient had a focal increase in the mesangium and its cellularity. He had a heavy (three plus) deposition of IgM in the mesangium. C1q was also detected in the mesangium. At present, we do not have EM facility at our center.

A diagnosis of primary IgM nephropathy needs exclusion of some conditions known to deposit IgM in glomeruli. These include diabetes mellitus, paraproteinemias, SLE, rheumatoid arthritis and Alport syndrome. Our patient did not have any of these conditions; however, he was diagnosed with Wilson’s disease.

The exact mechanism of IgM nephropathy is unknown. Some researchers have hypothesized; it may be secondary to complement cascade activation or immune complex mediated damage. Others suggest a defect in T-lymphocyte regulatory function or mesangial clearing of IgM from the glomerulus. There may be an environmental antigen trigger inducing increased production of IgM [2,5].

IgM nephropathy has never been reported with a primary liver disease. We performed a thorough PubMed search on this notion and found no case records. This prompted us to report this case and review the literature. Our patient was diagnosed with Wilson’s disease as shown by his laboratory parameters. He, however, did not have any other manifestations of the disease. Immunoglobulin A (IgA) nephropathy is well described with chronic liver disease; this hepatic-associated IgA nephropathy is considered the most common form of secondary IgA nephropathy. The mechanism is probably secondary to decreased hepatic clearance of immune complexes and increased deposition in glomeruli, aided by portal hypertension and shunting of blood into systemic vasculature. There have also been descriptions about impaired control of IgA production in patients with liver diseases [6]. The liver also plays a role in the removal of IgM [7]. Thus, the same mechanism as with hepatic-associated IgA nephropathy may be involved in the secondary IgM nephropathy encountered in our case. A few studies have reported an elevated level of IgM and IgM immune complexes in patients with IgM nephropathy [1].

IgM nephropathy is more common in children and adolescents, though it may occur at any age. Our patient was an adolescent male. The disease is slightly more common in males. The most common presentation is usually with nephrotic syndrome. Our patient had a similar presentation [2]. There is no consensus guideline on the treatment of the disease. Oral corticosteroids are the mainstay of treatment. However, the problem of steroid resistance is ever present. Various figures of steroid resistance have been reported with the greatest being 66% [1-2]. A study in Pakistani children revealed a steroid resistance of about 33% [8]. Our patient did not respond to steroids. He, however, responded well to intravenous cyclophosphamide with the resolution of proteinuria and edema. Some past researchers have used oral cyclophosphamide and response rates of 50% have been reported [2].

## Conclusions

We report the first known association between IgM nephropathy and Wilson’s disease. It is presently not clear if causation can necessarily be established. The mechanism may include defective IgM clearance by the liver or an altered metabolism of the antibody or immune complexes, as with hepatic-associated IgA nephropathy. Further studies are needed to elucidate the exact mechanism of this disease. We encountered steroid resistance in our patient, who initially responded well to intravenous cyclophosphamide and concurrent Wilson’s disease treatment.

## References

[1] Mokhtar GA ( 2011 ). IgM nephropathy: clinical picture and pathological findings in 36 patients. Saudi J Kidney Dis Transpl.

[2] Mubarak M, Kazi JI (2012). IgM nephropathy revisited. Nephrourol Mon.

[3] Connor TM, Aiello V, Griffith M, Cairns T, Roufosse CA, Cook HT, Pusey CD (2016). The natural history of immunoglobulin M nephropathy in adults. Nephrol Dial Transplant.

[4] Mubarak M, Lanewala A, Kazi JI, Akhter F, Sher A, Fayyaz A, Bhatti S (2009). Histopathological spectrum of childhood nephrotic syndrome in children in Pakistan. Clin Exp Nephrol.

[5] Hsu HC, Chen WY, Lin GJ, Chen L, Kao SL, Huang CC, Lin CY (1984). Clinical and immunopathologic study of mesangial IgM nephropathy: report of 41 cases. Histopathology.

[6] Alghamdi SA, Saadah OI, Almatury N, Maghrabi JA (2012). Hepatic-associated immunoglobulin- a nephropathy in a child with liver cirrhosis and portal hypertension. Saudi J Gastroenterol.

[7] Yan J, Vetvicka V, Xia Y, Hanikýrová M, Mayadas TN, Ross GD (2000). Critical role of Kupffer cell CR3 (CD11b/CD18) in the clearance of IgM-opsonized erythrocytes or soluble beta-glucan. Immunopharmacology.

[8] Kazi JI, Mubarak M, Malik SS (2010). Clinicopathologic study of IgM nephropathy in children presenting with idiopathic nephrotic syndrome in Pakistan. J Pak Med Assoc.

